# Variety of Ordered Patterns in Donor–Acceptor Polymer Semiconductor Films Crystallized from Solution

**DOI:** 10.1021/acsami.1c00079

**Published:** 2021-04-16

**Authors:** Shunpu Li, Jin Li, Youngtea Chun, Pawan K. Shrestha, Xin Chang, Mike Pivnenko, Daping Chu

**Affiliations:** † Centre for Photonic Devices and Sensors, University of Cambridge, 9 JJ Thomson Avenue, Cambridge CB3 0FA, United Kingdom; ‡ College of New Materials and New Energies, Shenzhen Technology University, Shenzhen 518118, China; § Department of Electronic Material Engineering, Korea Maritime and Ocean University, Busan 49112, South Korea

**Keywords:** crystallization, donor−acceptor polymer semiconductor, variety of ordered pattern

## Abstract

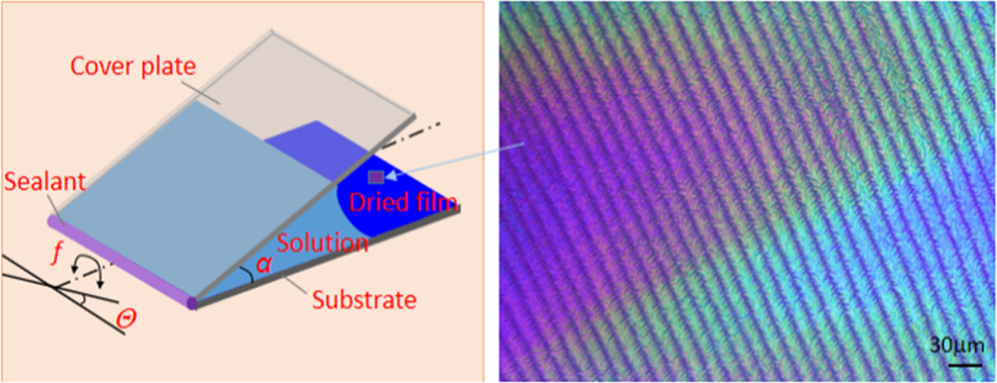

A huge challenge is to control the nucleation of crystallites/aggregates in the solution during polymer film formation to generate desired structures. In this work, we investigate crystallization of P(NDI2OD-T2), a donor–acceptor polymer semiconductor, with controlled solution flow along the contact line between the drying film and solution through a seesaw-like pivoting of samples during polymer drying. By controlling the pivoting frequency/amplitude, various types of line patterns can be observed: (I) an array of fishbone-like stripes oriented in the film-growth direction; (II) the pinning–depinning of contact line (PDCL)-mechanism-defined patterned wires along the contact line; and (III) periodic twined crystalline line pattern oriented in the direction of the contact line. The rich variety of pattern formation observed is attributed to the distinctiveness of the donor–acceptor conjugated polymer structure. The result measured from thin-film transistors made of the generated films/structures showed that the charge mobility of P(NDI2OD-T2) does not change much with the film morphology, which supports recent controversy over the charge-transportation mechanism of some donor–acceptor polymer semiconductors.

## 1 Introduction

Crystallization from the liquid is one of the most ancient techniques known to mankind, for instance, ice making and salt purification. It has been studied extensively in the past 100 years and crystal growth still plays an increased role in modern science and technology. Organic crystal growth or aligning molecule chains with a solution process have been the subjects of many studies in different domains, like proteins/DNA,^1−3^ liquid crystals,^4^ and nonlinear optical crystals.^5^ A very new area in this field is polymer semiconductor thin-film crystallization from a solution as this may lead to important consequences on the performance of optoelectronic devices because charge-carrier mobility is strongly influenced by the packing of the conjugated molecule chains.^6−8^ For more than a decade, research has primarily focused on increasing the long-range order and the crystallinity of polymers as a strategy to improve the charge-transport properties. The charge-carrier mobility has increased by orders of magnitude through the design and synthesis of semiconductor polymers, which can form a highly ordered phase and this favors charge hopping between stacked chains.^9−11^ However, this concept may not always be true as there is a controversy about the charge-transportation mechanism in a number of donor–acceptor (D–A) semiconductor polymers. Some researchers suggested that for certain D–A polymers, the crystallinity does not increase the charge mobility, instead, such materials have a high tolerance for disorder by allowing more efficient intra- and intermolecular charge-transport pathways,^12−14^ while other researchers have obtained a contrary result.^15,16^ To solve this dispute, further experimental evidence is required. A straight way to address this problem is to generate a molecule-oriented or crystalline film and investigate its charge-transportation property. On the other hand, the crystallization behavior of D–A semiconductors itself is an interesting research topic as such polymers possess distinctive folding characters due to large chain stiffness and intermolecular stacking properties, i.e., (D–A)/(A–D), (D–A)/(D–A) etc., alternative molecule sequence overlaps in the materials.^14,17^ Meniscus-guided deposition is a popular technique to fabricate organic films with oriented molecules from solutions.^18,19^ Many studies involved mechanically pulling of substrates or solutions, which force polymer chains to align.^20,21^ Other methods, for instance, space restriction or textured substrates can also be used to guide molecule alignment with the liquid process.^8,22−24^

With the motivation of the “Czochralski technique”, where a relative motion between a growing crystal and liquid is introduced by rotating the crystal or crucible to avoid crystal nucleation in the liquid at the crystallization front, in this work, we attempt to modify the “meniscus-guided deposition” with a seesaw-like pivoting of drying samples. Our experiment shows that the introduction of solution flow along the contact line can restrict polymer nucleation at the front of drying and regular patterned P(NDI2OD-T2) fishbone-like stripes oriented in the growth direction are obtained. By modifying the experimental parameters, we were able to generate two other types of P(NDI2OD-T2) patterns formed by different mechanisms that demonstrate the variety of pattern formation in the D–A-conjugated polymers. In addition, thin-film transistors (TFTs) were fabricated with the generated films/patterns and the result supports the controversy over the charge-transportation mechanism in D–A polymer semiconductors.

## 2 Results and Discussion

**Figure 1:**
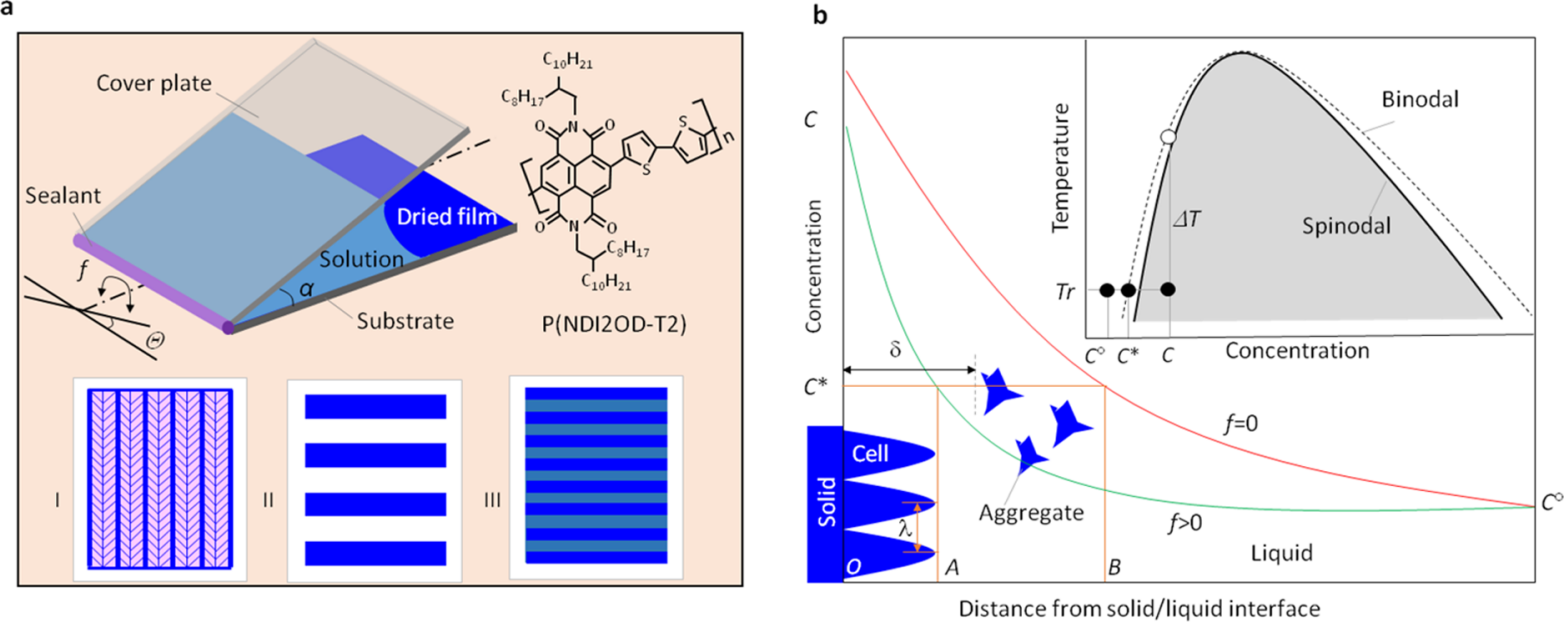
Schematic illustration of the experimental setup and the working principle. (a) Schematic drawing of the sample-pivoting setup and three typical patterns generated in the P(NDI2OD-T2) film. (b) Schematic drawing of the concentration-distribution change in the solution caused by the sample pivoting and the cellular-shaped crystallization front induced by a narrowed undercooling region. The inset of (b) shows a schematic binary phase diagram of the polymer solvent.

Figure 1 shows a schematic drawing of the experimental setup used in this work. A P(NDI2OD-T2) (*M*_n_ = 50–100 kDa) toluene solution (2.5 wt %) is confined in a wedge-shaped space formed by a substrate and cover plate (for most samples, the open-angle α = 5° unless indicated otherwise). The pivoting of the solution-loaded samples was realized on a platform (Quantum Scan-30 Galvanometer Scanner) with tuned frequency and amplitude (Figure 1a). The sample pivoting can reshape the polymer concentration distribution in the solution at the vicinity of the contact line. Figure 1b shows the principle of the experiment. At static conditions, the polymer concentration distribution in a solution depends on a number of factors, like solid–liquid interface diffusion and surface-tension/temperature-induced convection, etc., and is illustrated with the red curve that is similar to the impurity distribution in the melt next to the crystallization front during crystal growth.^25^ The sample pivoting forces the solution to flow and the concentration distribution becomes narrower (green curve). The inset of Figure 1b shows a schematic phase diagram of a polymer-solvent, where binodal and spinodal curves are marked by dashed and solid lines, and *C* is the polymer concentration.^26^ If polymer concentrations/temperature are located in the spinodal region, the homogeneous solution is unstable against a small fluctuation in density or concentration, and the solution separates into two phases with a well-defined size (i.e., spinodal wavelength λ_SN_). In bulk solution, the polymer concentration *C*^0^ is outside of the binodal curve, which defines a stable solution. At the vicinity of the contact line, the solution concentration *C* is in the spinodal region and crystallization occurs. Δ*T* is undercooling, which defines a driving force for nucleation of crystallites in the solution. Δ*T* is caused by the concentration deviation from the critical concentration C* (see Figure 1b, when *C* = C*, Δ*T* = 0), which is similar to the “constitutional undercooling” in metallurgy.^27^ Under the experimental condition (room temperature), when *C* > C*, the undercooling Δ*T* > 0 and crystallization in the liquid can happen. The C* and the concentration profile in the solution define an undercooling region in which Δ*T* > 0 at all points (see OA or OB). The sample pivoting changes the solution-concentration profile from the red to the green curve, and consequently the length of the undercooling region changes from OB to OA. If the OA is shorter than a characteristic polymer diffusion length δ, the polymer chains can be ferried to the solid/liquid interface by diffusion and attach to the crystallization front. In contrast, for static samples, when nucleation of crystallites/aggregates happens in a wider undercooling region (OB), which is longer than δ, new crystals or aggregates are formed in the solution and the dried films are polycrystalline or amorphous.

**Figure 2:**
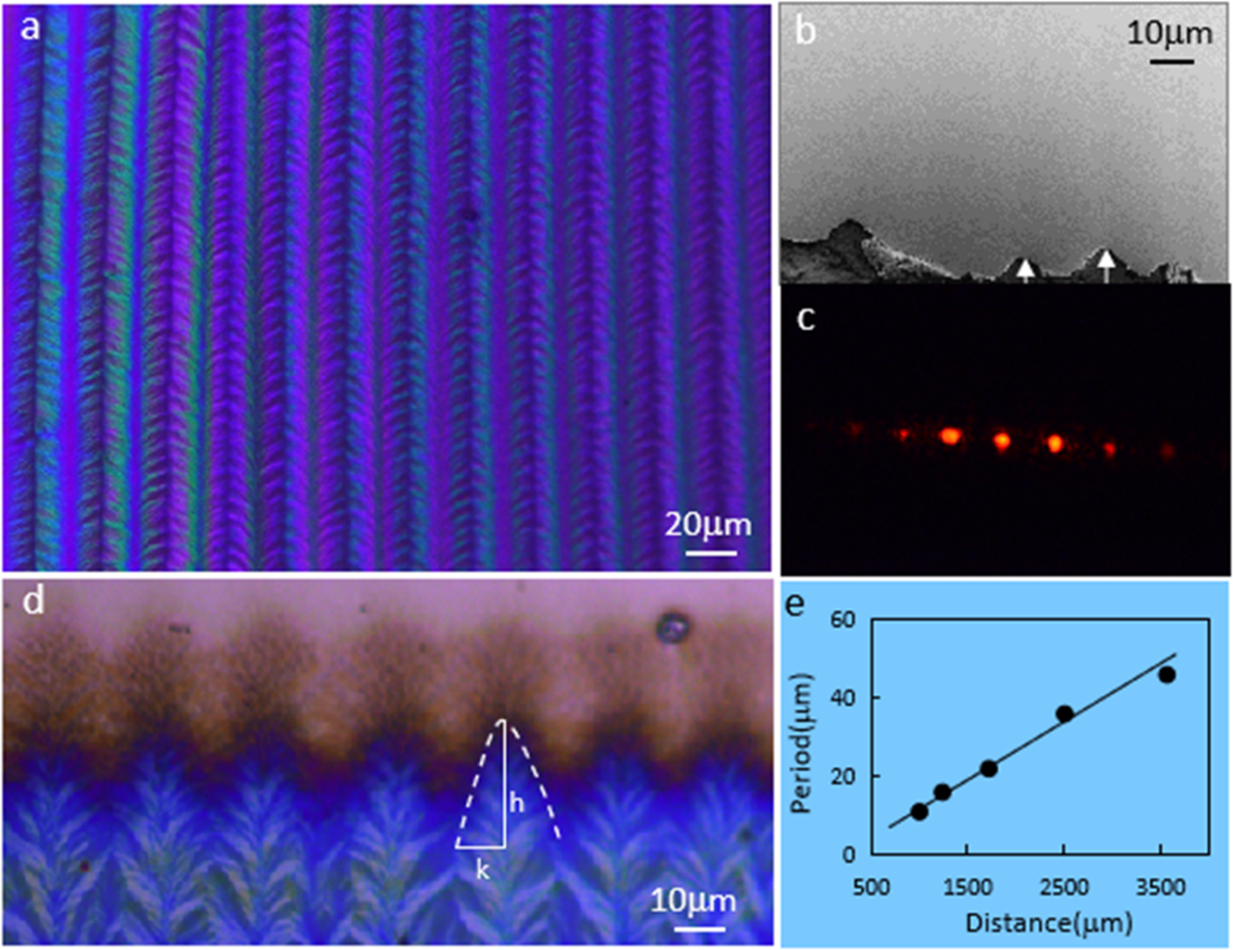
Microstructures of the generated fishbone patterns. The optical microscopic image of the typical fishbone pattern (*f* = 6 Hz, Θ = 2°) (a), the different color contrast may be caused by the slight difference of crystallization conditions at different locations of the drying front; the SEM image of such fishbone pattern shows a very little topographic contrast (b); (c) laser diffraction spot pattern to show that the regular structure is over a large area; (d) cellular-shaped crystallization front generated by quenching the growth by suddenly removing the solution (*f* = 6 Hz, Θ = 2°); and (e) fishbone widths (period) taken at different locations measured from the sample edge where the substrate and cover plate are bonded. The sample used for this measurement is the same as used for (a).

Figure 2a shows an optical image of an oriented fishbone structure obtained by sample pivoting with frequency *f* = 6 Hz and angle amplitude Θ = 2°. Figure 2b shows scanning electron microscopy (SEM) analysis of such a fishbone structure, which shows very little topographic contrast and this has been confirmed with atomic force microscopy (AFM) measurements (Figure S1). We speculate that the fishbones have a crystalline structure with the amorphous phase filled in between them, and both crystalline and amorphous phases compose a uniform film. The domain size with regular fishbone structures spans over several millimeters and the pattern regularity can be revealed by optical diffraction (Figure 2c). The film-growth speed and film thickness were measured to be 4.5 μm/S and 110 nm. To reveal more information about the crystallization process, the crystallization front has been quenched by sudden removing of solution during crystallization with a liquid-absorbing fibber material. One can see that the growth front has a cellular morphology, extending into an undercooled solution during the crystallization (see Figure 2d). The regularity of the structure can be explained by spinodal precipitation,^26^ and it interprets an initial periodic perturbation, which defines the fishbone period, and the cellular morphology is caused by the growth of the structure in an undercooled solution. The detailed structure of the fishbone is related to Mullins–Sekerka instability.^28,29^ The cell growth process is balanced by the stability-favor factor (surface energy) and instability-favor factor (undercooling). Under a given experimental condition, molecules are deposited onto the cell front in a specific way (face-on or edge-on) to adjust the surface energy. The spinodal process defined length scale λ_SN_ is
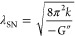
1where *G*″ is the second derivative of the free energy of a solution system with respect to composition and *k* is the “square gradient” parameter, accounting for changes in free energy arising from concentration gradients.^26,30^ By taking *k* ∼ 0.4 (Å·mol·cm^–3^) and −*G*″ ∼ 10^–7^ (mol·cm^–3^) (Note 1 of Supporting Information and ref (30)), one can estimate λ_SN_ ∼ 1.7 μm, which is in the same order of magnitude as the experiment.

We have found that the periodicity of the fishbone structure decreases in the growth direction caused by the reduction of the height of the meniscus (Figures 2e and S2). Research on spinodal precipitation in confined geometry has shown that the size of the precipitated phase decreases with the reduction of spaces.^31^ The periodicity of the pattern is self-adjusted by branching, joining, and growth termination to optimize the λ_SN_ value (Figure S3). Branching is a frequently observed process as the film is grown in the direction with gradually narrowed space and the period of structure reduces accordingly. However, joining and termination are “defects” caused by local environmental variations.

**Figure 3:**
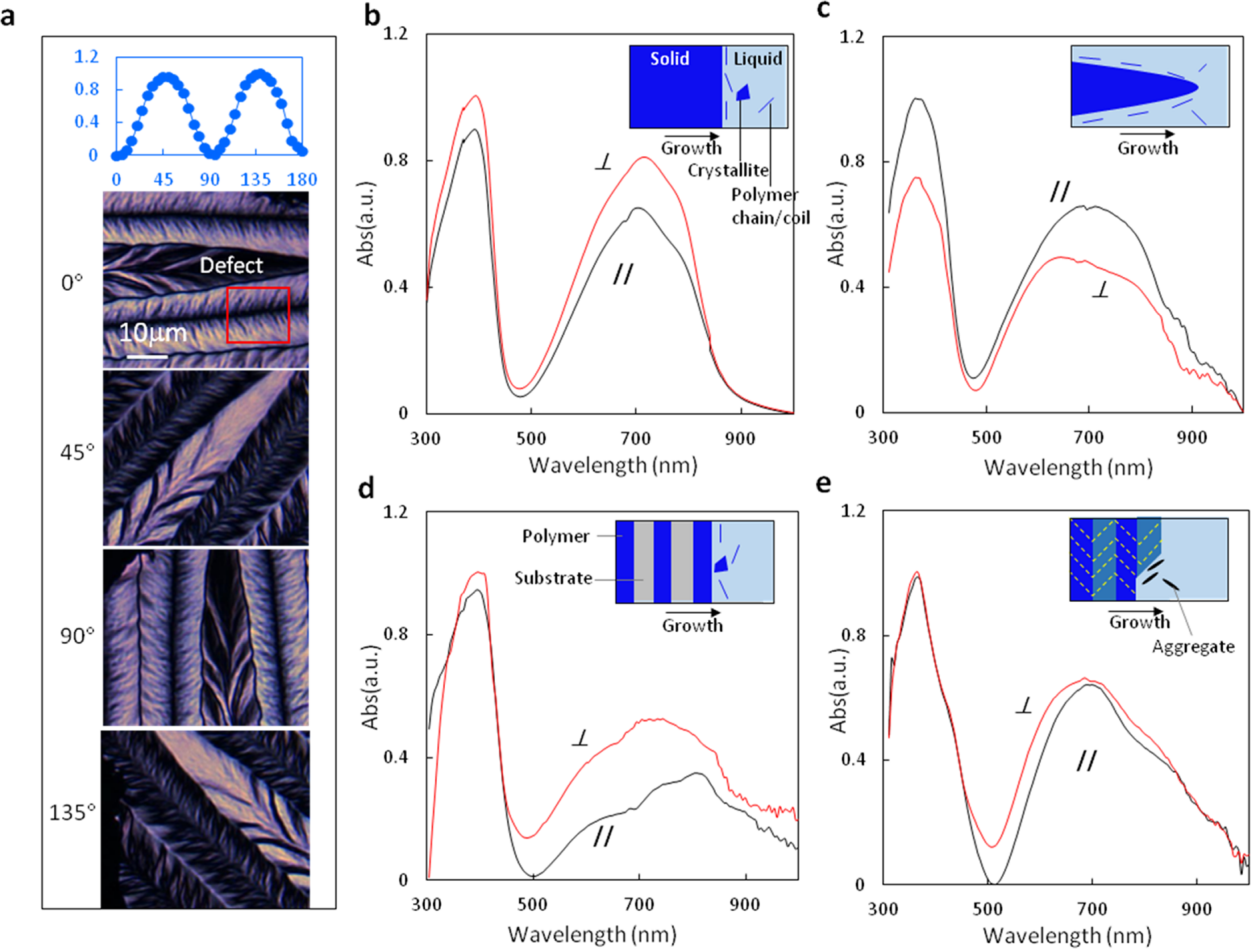
PM and PAS measurements from samples generated at different conditions. (a) PM images taken from a sample with a fishbone structure (*f* = 6 Hz, Θ = 2°) at different rotation angles. Angle dependence of normalized brightness taken from a tracking point on the sample is also shown (top panel); (b) PAS measurement result for the unpatterned film (*f* = 0); (c) PAS measurement result from a sample with a fishbone pattern (*f* = 6 Hz, Θ = 2°); (d) PAS measurement result from a sample with the PDCL-mechanism-defined line patterns (α = 20°, *f* = 6 Hz, Θ = 2°); and (e) PAS measurement result from a sample with a twinned crystalline structure (*f* = 0); the result shows a similar absorption property for the light polarization axis that is parallel to both the film-growth direction and contact line direction. This indicates that the preferential polymer-chain orientation is in the direction that can be equally decomposed into the film-growth direction and the contact line direction. The symbols “⊥” and “//” in the figures denote that the directions of the light polarization axis are perpendicular and parallel to the film-growth direction.

The amorphous regions between the fishbones are mechanically weak regions, as shown by the sunken regions in the tweezer-scratched area in Figure 2b (indicated with white arrows). Dewetting can initiate from the place between two neighbored fishbones and an array of separate lines can form when the fishbone patterns are generated on less wetted substrates (Figure S4). The conformation of the polymer chains in the fishbones has been analyzed using a polarized microscope (PM) and a strong birefringence has been observed (Figure 3a). The observation and photograph were focused around a defect (a terminated fishbone) to trace the studied location. One can see a strong brightness contrast between the center and edge areas of the fishbone (red frame in Figure 3a), which is induced by an orientation variation of the polymer chain in the same fishbone. We propose a “ladder” conformation of polymer chains (sequentially arranged crystallites formed by folded chains) in the fishbone, which is further confirmed by polarized UV–vis absorption spectroscopy (PAS). If the dipole moment of the main electronic transition is oriented along the conjugated backbone, the maximum absorption of PAS is expected when the polymer chains align with the light polarization axis because the coupling strength (*g*) between the transition dipole moment (μ) and the local electrical field (*E*) can be expressed by *g* = **μ**·**E**.^32,33^ We have first measured PAS from an unpatterned film formed by crystallization at static condition (*f* = 0). Images of such an unpatterned film and quenched growth front are shown in Figure S5. The maximum absorption was found when the light polarization axis was perpendicular to the film-growth direction (i.e., the direction along the solid/liquid contact line), which indicates that the polymer chain is preferentially aligned along the crystallization front (Figure 3b). The preferential chain orientation in the unpatterned film is also supported by PM measurement (Figure S6). However, the measured PM contrast is only about 5% of that measured from a fishbone. This could be caused by only a small portion of the chain alignment along the contact line, while the majority of the film is formed by random stacking of aggregates formed in the solution (inset of Figure 3b). The preferential chain alignment along the drying line for the D–A polymer film has been observed previously.^34^ The PAS measured on the fishbone structure shows that the maximum absorption happens when the light polarization axis is parallel to the film-growth direction (Figure 3c). This indicates that the preferential chain orientation is in the film-growth direction and agrees with our anticipation: the molecule backbone aligns along the local surface of the cellular front (Figure 3c). The ratio of the PAS strength taken parallel and perpendicular to the growth direction should be proportional to the ratio of the long axis/short axis of a front “cell” (*h*/*k* in Figure 2d). If *h* > *k*, which is the case in our experiment, the absorption signal is dominant when the light polarization axis is parallel to the film-growth direction.

**Figure 4:**
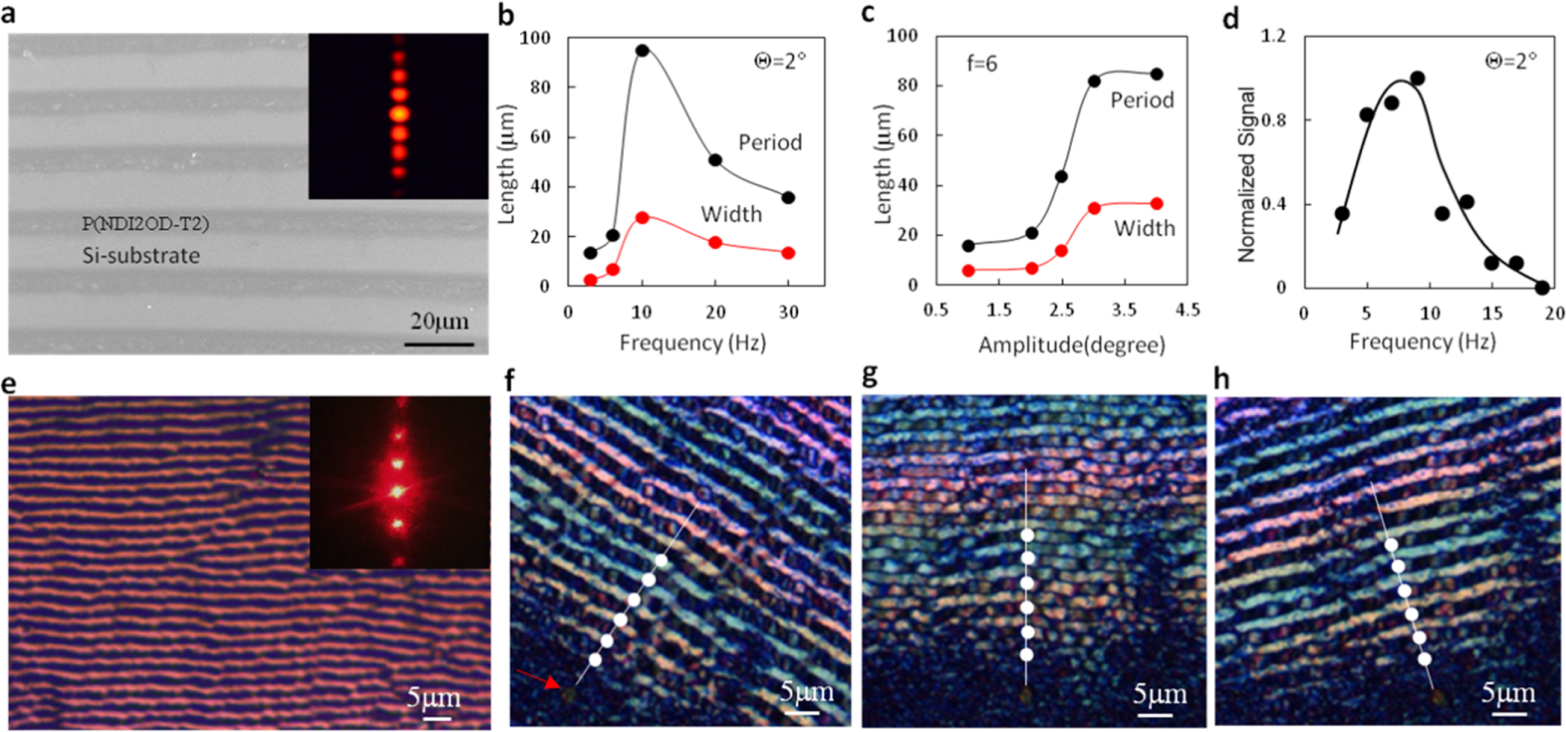
Characterization results of PDCL-mechanism-defined line patterns and twinned crystalline patterns. (a) SEM image of PDCL-mechanism-defined line patterns (α = 20°, *f* = 6 Hz, Θ = 2°); (b, c) pivoting frequency and amplitude dependence of the line width/period for the PDCL-mechanism-defined line patterns; (d) frequency dependence of measured solution-vibration strength (see Figure S9); (e) optical image of the twinned crystalline structure and laser diffraction from the pattern; and (f–h) PM images of the twinned structure taken at different rotation angles, where the string of white dots indicates the contrast change of the pattern [the dots are marked on bright stripes in (f) and they are located on the dark stripes in (h)]. The red arrow in (f) indicates a reference mark on the sample.

We have also found that with increasing the open angle α, patterns of separated lines oriented along the contact line are formed (Figure 4a). The width and period of the separated lines can be tuned many times by varying the pivoting frequency and amplitude (Figures 4b,c and S7). The maximum line width/period was observed when the frequency was around 10 Hz, while a drastic enhancement of width/period with a pivoting amplitude was found between 2° and 3° and then saturated. Such self-assembled lines have been observed previously and a repeated pinning–depinning of contact line (PDCL) mechanism was proposed;^35,36^ however, no effect of mechanical perturbation has been inspected before. We explain the line width/period change as that the sample pivoting induces a liquid surface vibration, which makes the contact-line depinning less sensitive to the receding angle variation with solvent evaporation (Figure S8). Our experiment showed that the maximum amplitude of liquid surface vibration is found at the frequency at 9 Hz (Figures 4d and S9), which agrees with our microstructure examination and resonance behavior of the liquid bridge study.^37^ Such PDCL-mechanism-defined array of lines contains polymers with a preferential chain orientation along the line orientation, as proved by PM (Figure S10) and PAS observation. In the PAS, the maximum absorption is observed when the light polarization axis is perpendicular to the film-growth direction (i.e., parallel to the solid/liquid contact line) (Figure 3d).

There are many factors that could affect the pattern/film formation: (1) During the formation of PDCL-mechanism-defined line patterns, film dewetting from the substrate plays a significant role. The trenches of the structure generated by the PDCL process are the natural weak linkage for dewetting to start. (2) The elastic property of the polymer material is a factor to maintain the regularity of the generated patterns and uniformity of thin films as drying is processed under sample vibration. (3) The rheological characteristics of the polymer solution can play a significant role during the polymer crystallization,^38,39^ as the polymer-chain conformation in the solution is modified during the sample pivoting. The solution flow induces a shear stress at the drying front, which may force the polymer chain to align along the drying front locally. Once the crystallization happens, the polymer chains will relax in the freshly formed film, and this will influence the film stability. (4) Thermal fluctuation could also have some effect on the stability of the formed films, as local strain can be influenced by a small temperature fluctuation.^40^ Apart from the abovementioned fishbone- and PDCL-defined patterns, we have observed another type of regular line pattern in the P(NDI2OD-T2) films. Previous research on casting P(NDI2OD-T2) films from solution showed that if the solution contains sufficient aggregates, birefringence patterns can appear in dried films.^16^ From the above discussion (see Figure 1b), we know that if the sample-pivoting frequency is low, preaggregation may happen in the solution near the crystallization front. This means that we may find a regular periodic birefringence pattern in a continuous film crystallized under a low pivoting frequency, although unpatterned films are formed normally. Figure 4e is the observed structure, which shows periodically patterned stripes oriented along the contact line taken under a polarized optical microscope. Such regular birefringence is arisen from an embedded pattern of the polymer chain with a zigzag configuration, as confirmed with PM and PAS measurements (Figures 4f–h and 3e). We would like to address that the repeatability of obtaining such a patterned twin structure is poor, and the reason might be the difficulty to obtain aggregates with uniform size. Such pattern is rarely obtained in polymer films and has been found a few years ago in PII-2T, another D–A conjugated polymer semiconductor, film crystallized on ionic liquid as a dynamic template.^34^ The mechanism of the pattern formation is not clear, which requires further investigation. We propose a possible model to explain the twinned structure formation (detailed in Figure S11). In this model, the depinning is initiated locally to form a kink on the contact line and the kink propagates along the original pinning line to complete one step of the depinning and repining. The process is repeated immediately after each completion of the previous depinning step. The aggregates of P(NDI2OD-T2) in the solution are elongated along which the molecule chains are oriented and folded.^16^ In pace with the propagation of the kink, the aggregates are deposited onto the kink and get aligned there. When the kinks of two neighbor-depinning steps propagate in the opposite directions, a twined pattern can form.

**Figure 5:**
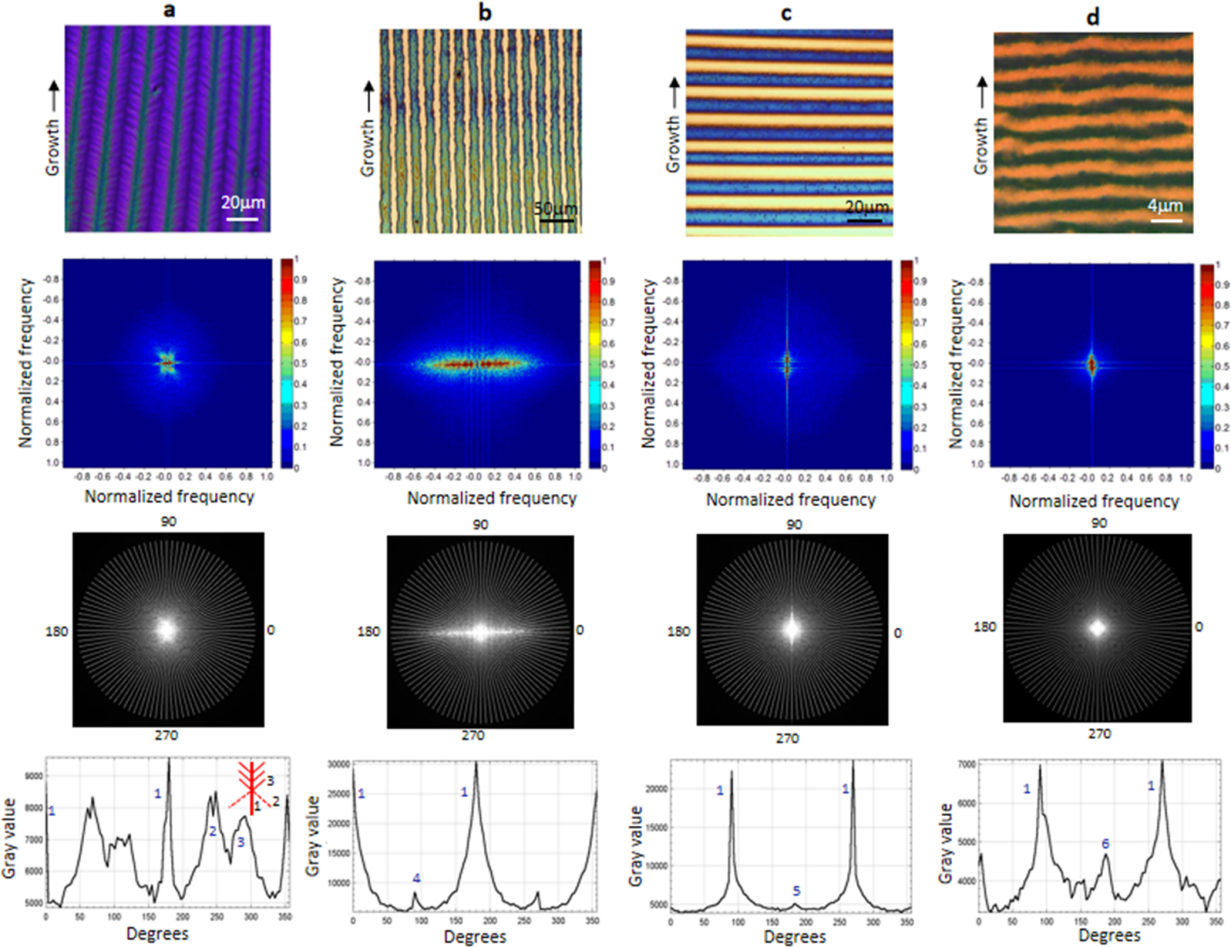
Pattern analysis result with 2D fast Fourier transformation (2D FFT) performed on the images of the four types of oriented structures: (a) array of fishbone, (b) dewetted fishbone, (c) PDCL-mechanism-defined lines, and (d) twinned crystalline pattern. The top panel: optical structures of analyzed patterns; the second panel: frequency spectra generated with 2D FFT; the third panel: various directions in which the values of gray levels are summed; and the bottom panel: the plotting of spectral intensity distribution with different angles.

Figure 5 shows pattern analysis result with two-dimensional fast Fourier transformation (2D FFT) performed on the images of the four types of oriented structures (array of the fishbone (a), dewetted fishbone (b), PDCL-mechanism-defined lines (c), and twinned crystalline pattern (d)) using MATLAB software with an FFT function. After the creation of the frequency spectrum under the normalized frequencies (second panel), a zero-frequency-centered circle area with a proper radius is chosen from the frequency spectrum to sum the values of gray levels for various directions spanning from 0 to 2π with the interval of 2π/100 (third panel). The plotted spectral intensity distribution with different angles offers a quantitative description of the structural property (bottom panel).

For all four cases, there are sharper peaks (indicated by 1) separated by 180°, which signifies a primary one-dimensional orientation as expected. In the case of the fishbone structure (Figure 5a), two satellite broadened peaks (indicated by 2 and 3) between the primary peaks reflect the oriented substructure on both sides of a fishbone (inset of the bottom panel in Figure 5a). For the dewetted fishbone (Figure 5b), the peaks 2 and 3 disappeared and a new small peak 4 emerged. This is explained by an elastic relaxation of the polymer material and mechanical tearing-induced feature that is oriented perpendicularly to the primary fishbone orientation during dewetting. A similar tearing mechanism can be used to explain the peak 5 for the PDCL-mechanism-defined lines in Figure 5c. The peak 6 in Figure 5d is explained by the existence of high density of micrometer-sized domains in each stripe, as shown in Figure 4f–h.

**Figure 6:**
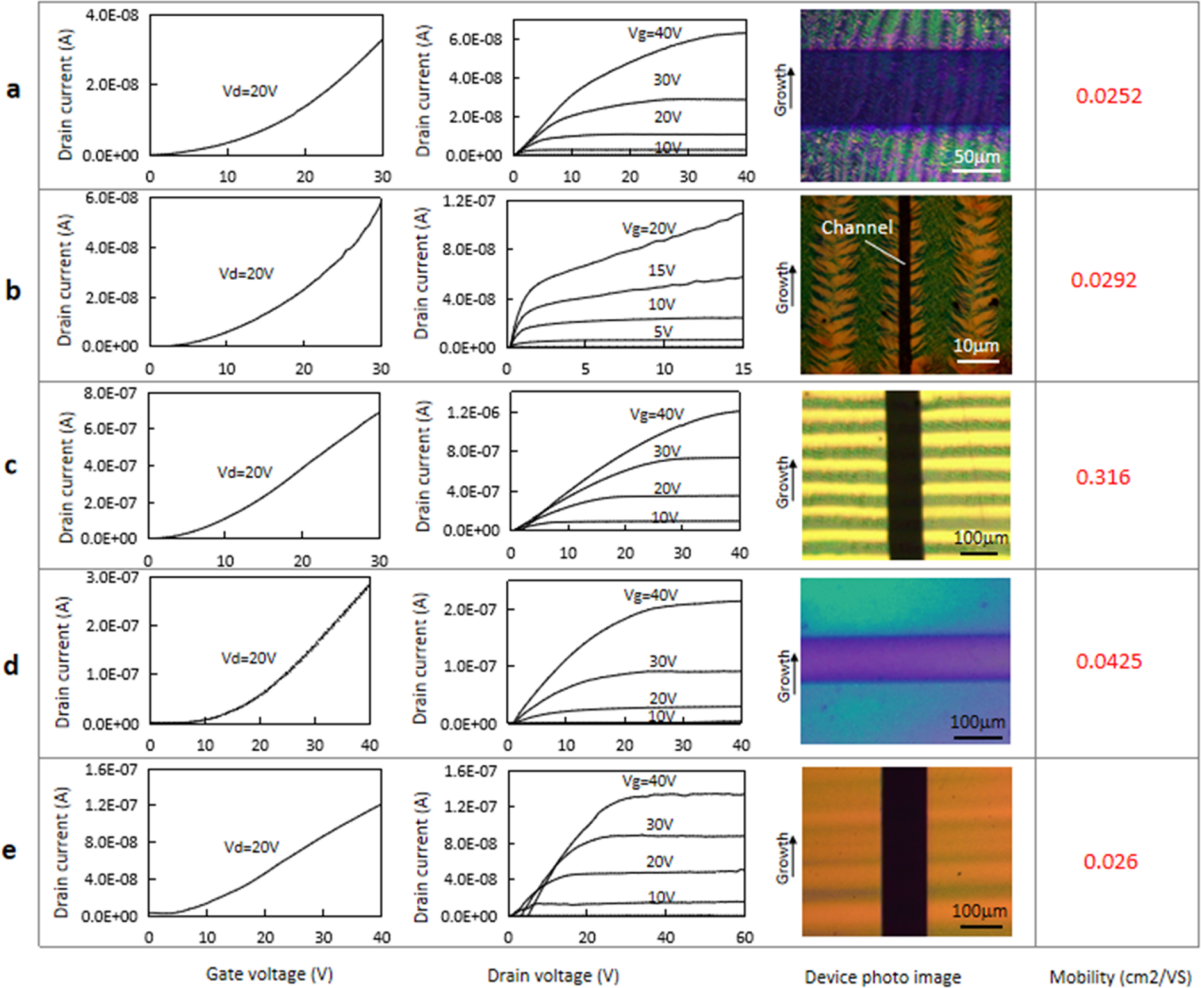
Test results of TFTs made of P(NDI2OD-T2) film/patterns created under different conditions. (a) TFT with fishbones stride over the channel; (b) TFT with a fishbone oriented along the channel; (c) TFT with patterned semiconductor lines formed by the PDCL mechanism; (d) TFT made from the unpatterned film with the growth direction perpendicular to the channel; and (e) TFT made of unpatterned films with the growth direction parallel to the channel.

We have also fabricated thin-film transistors with the P(NDI2OD-T2) films/structures created under different conditions (Figure 6). All TFTs were fabricated with the top-gate configuration on glass or SiO_2_(300nm)/Si substrates with predefined Au source–drain electrodes. After the creation of the semiconductor films with required patterns, the samples were annealed at 140 °C for 6 h in a nitrogen atmosphere. Then, 700 nm poly(methyl methacrylate) (PMMA) dielectrics layer was spin-coated from a butyl-acetate solution and dried at 80 °C for 30 min. Finally, a 100 nm Al film was deposited at the top of PMMA dielectrics through a shadow mask as a gate electrode. TFTs were tested at ambient conditions using an Agilent 4156C semiconductor analyzer. The field-effect mobility μ was estimated with the formula^41^
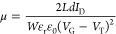
2where *W* and *L* are the channel width and length; ε_r_, ε_0_, and *d* are the relative permittivity, vacuum permittivity, and thickness of the dielectrics; and *I*_D_, *V*_G_, and *V*_T_ are the drain current, gate voltage, and threshold voltage.

Figure 6a shows the result of TFT with fishbones stride over the channel, while Figure 6b is that from the TFT with a fishbone oriented along the channel; Figure 6c shows the result of the TFT with patterned semiconductor lines formed by the PDCL mechanism; Figure 6d,e shows the results from TFTs made from unpatterned films with the growth direction perpendicular and parallel to the TFT channels, respectively. The measured charge mobility in the fishbone structure and the crystallized uniform film are in the range of 0.026–0.042 cm^2^/V·S, which does not show an obvious conduction anisotropy. This indicates that the charge mobility of P(NDI2OD-T2) does not change much with material crystallinity and molecule orientations in the crystallized film. This result supports the controversy over the charge conduction mechanism for some A–D-type polymer semiconductors, as some researchers claimed previously: certain A–D-type polymer semiconductors have a high tolerance for disorder by allowing efficient intra- and intermolecular charge transport.^13,14^ From the low charge mobility and isotropic conduction property of the fishbones, one can speculate that the crystals contained in the fishbone are fold-chain crystals. The obtained higher charge mobility for the PDCL-mechanism-defined lines can be explained by film-compression-induced molecule stretching along the lines caused by dewetting. The result obtained here shows that extended polymer chains are favorable for charge conduction, while the crystallization of the polymer does not benefit in terms of improving charge mobility.

## 3 Conclusions

In summary, we have observed that the P(NDI2OD-T2) semiconductor can possess very rich patterned structures with controlled sample-pivoting during crystallization. Three types of ordered patterns were observed: (I) patterned fishbones oriented in the film-growth direction and the polymer chains (or folded chains) adopt a ladder configuration; (II) pinning–depinning of contact line (PDCL)-mechanism-defined patterned lines oriented in the direction of the contact line and the polymer chains are preferentially aligned in the same direction; and (III) the periodic birefringence line pattern oriented in the direction perpendicular to the film-growth direction. We speculate that in all cases, the polymer chains are locally aligned along the front line of crystallization. The morphology of the crystallization front line varies with the conditions of crystallization. The TFT result showed that the charge mobility of P(NDI2OD-T2) does not change much with material crystallinity and molecule packing, which agrees with some previous research on D–A polymer semiconductors. This work is mainly focused on the pattern formation property in a D–A polymer semiconductor film; a detailed investigation of molecule orientation and intermolecular packing with more sophisticated techniques, such as 2D grazing incidence wide-angle X-ray scattering (GIWAXS) and two-dimensional nuclear magnetic resonance (2D-NMR), etc., are highly recommended in future work.

## 4 Experimental Methods

The pivoting samples used in this work were made via solution confinement in wedge geometry as many stable liquid bridges are formed with such geometry. For sample preparation, solutions were introduced using a micropipette into the wedge-shaped spaces formed with substrates (12 mm × 12 mm) and glass cover plates (12 mm × 12 mm) with required open angles. The joining of the substrate and cover plate can be made with epoxy or scotch tape. The substrates were treated with O_2_ plasma (100 W for 5 min) to increase the binding strength of the generated patterns/films with the substrates. The pattern generation was carried out at ambient conditions in a chemical fume hood. For TFTs with a longer channel length (>50 μm), the source–drain electrodes were fabricated by shadow mask deposition of 50 nm thick Au and 20 nm thick Cr as an adhesion layer; while for TFTs with a smaller channel length, the electrodes were defined by optical lithography and subsequent lift-off use Au(50 nm)/Cr(20 nm) films. The AFM and SEM analysis were performed on a Nanoscope III and LEO GEMINI 1530, FEG-SEM system. The PAS was measured using the JASCO ARSN-733 absorption spectrometer with an installed polarizer. The PM experiments were done using a BX51 polarizing microscope (OLYMPUS). To compare the PM results measured from the fishbone samples and unstructured samples, both types of samples were measured at the same conditions (incident light intensity, objective lens, etc.). The relative optical signal strength refers to the ratio of the maximum brightness measured from the two types of samples.

## Supporting Information Available

The MFM image; the fishbone structure taken at different positions; typical defects observed in the fishbone arrays; the array of separated lines formed from dewetting of the fishbone pattern during growth; the morphology of the quenched front of the film grown at zero frequency; PM images; the PDCL mechanism; the schematic illustration of delayed depinning of the contact line when the sample is dried under pivoting conditions; the schematic drawing of the setup for measuring the liquid vibration strength; polarized microscopic images of the PDCL mechanism; and the schematic illustration of the proposed possible “kink propagation” mechanism (PDF)

## Notes

The data that support the findings of this study are available from the corresponding author upon request.
