# Supplementary material for: Variety of Ordered Patterns in Donor–Acceptor Polymer Semiconductor Films Crystallized from Solution
Source: ACS Appl Mater Interfaces. 2021 Apr 16;13(16):19055–63. doi: 10.1021/acsami.1c00079 (PMC8153537; doi:10.1021/acsami.1c00079)
Supplement: Supplementary file 1 — am1c00079_si_001.pdf [file am1c00079_si_001.pdf]

Supporting Information

Variety of Ordered Patterns in Donor-Acceptor Polymer Semiconductor Films Crystallized From Solution

Shunpu Li,[§, #, †] Jin Li,[§, †] Youngtea Chun,[§,⊥] Pawan K. Shrestha,[§] Xin Chang,[§] Mike Pivnenko,[§] Daping Chu [§,*]

[§] Centre for Photonic Devices and Sensors, University of Cambridge, 9 JJ Thomson Avenue, Cambridge CB3 0FA, United Kingdom

[#] College of New Materials and New Energies, Shenzhen Technology University, Shenzhen 518118, China

[⊥] Department of Electronic Material Engineering, Korea Maritime and Ocean University, Busan, 49112, South Korea

[†] These authors contributed equally to this work.

[*] Correspondence author (Email: dpc31@cam.ac.uk)

Note 1: Evaluation of k value

We use published work about polymer spinodal decomposition to estimate k value. We have fitted curve of Fig. 4a in Ref 25 with low concentrations C (less than 15wt-%) and we have obtained an expression $k=0.03C^{-0.7}$. Thus, we can extend the result to lower concentrations which is not included in the reference and we have k=0.4 for C=wt-2.5%.

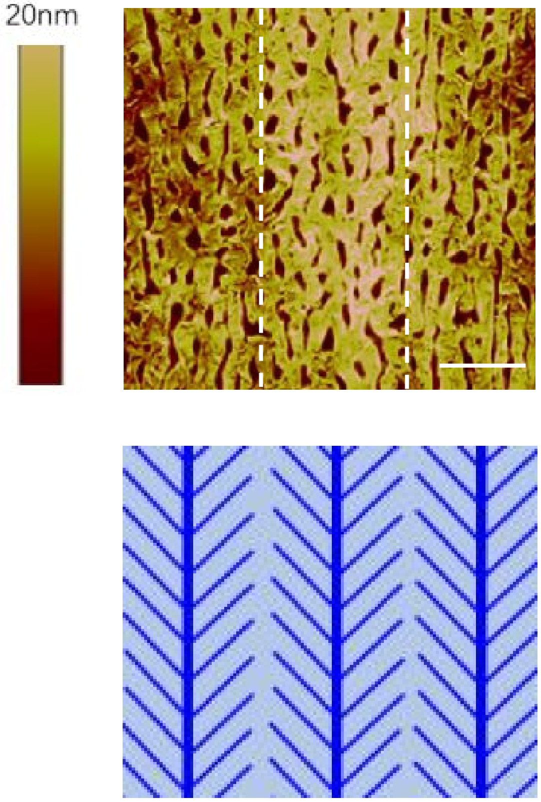

Fig.S1: (a) MFM image of P(NDI2OD-T2) film with fishbone structure; (b) Schematic fishbone

pattern corresponding the AFM image in (a) . The three fishbones are marked with white dotted lines in (a).

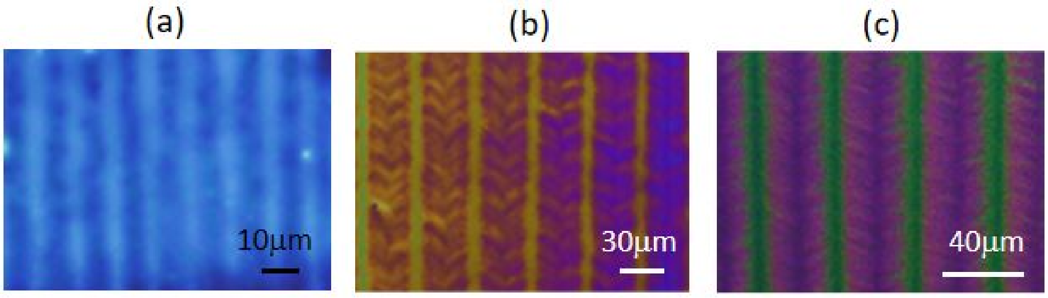

Fig.S2: Fishbone structure taken at different positions (x) measured from the sample edge where the substrate and cover-plate are bonded. (a) Image was taken at x=1000μm; (b) Image was taken at x=2550μm; (c) Image was taken at x=3300μm.

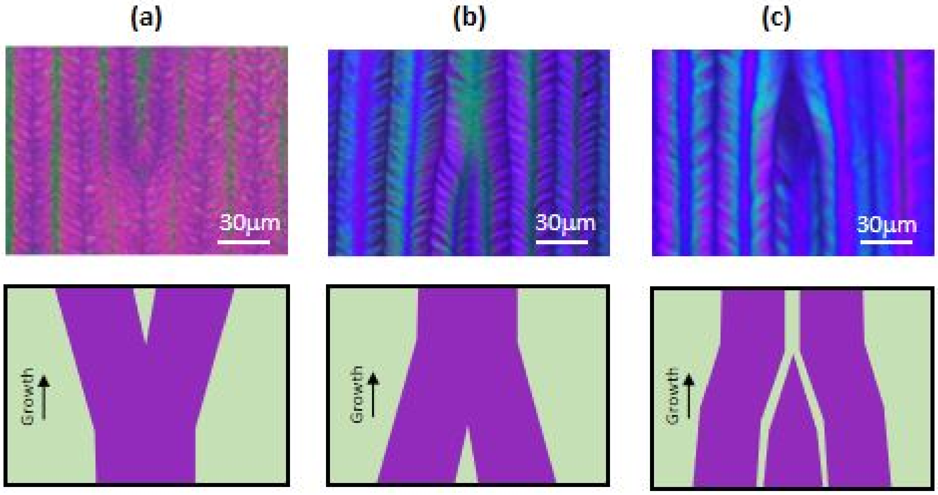

Fig.S3: Typical defects observed in the fishbone arrays and schematic illustration of the branching (a), joining (b), and termination (c) processes. The branching is frequently observed process as the film is grown in the direction with gradually narrowing space and the period of the

structure is reduced. While, the joining and termination are real defects caused by local

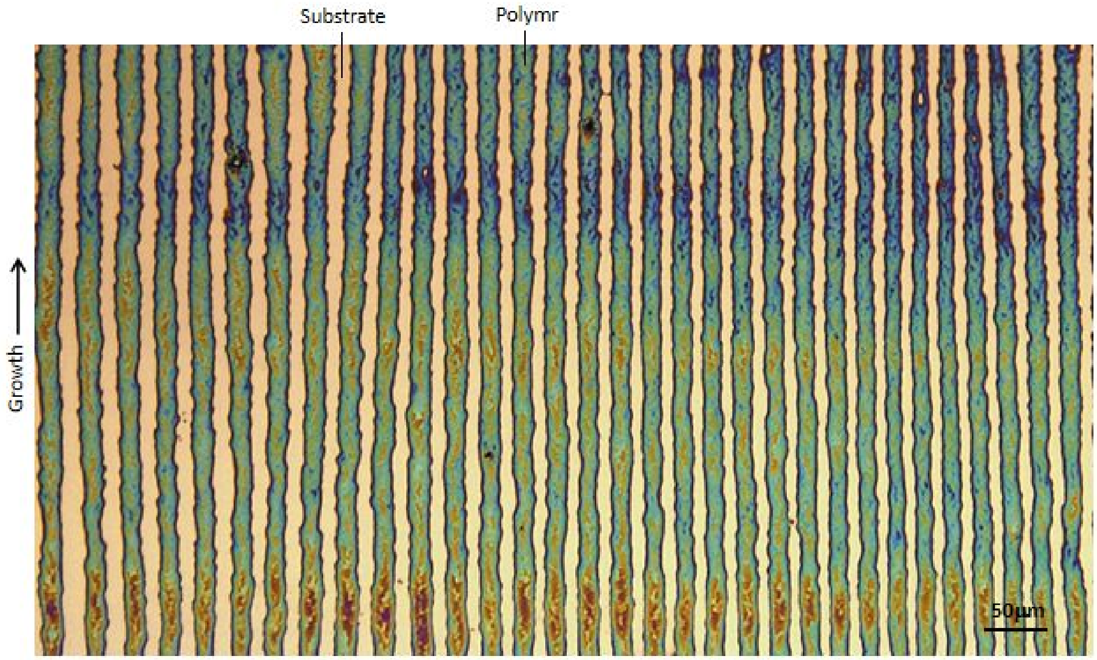

environment variations and are rarely observed.

Fig.S4: Array of separated lines formed from dewetting of fishbone pattern during growth. The pattern was grown on substrate without O_2 plasma treatment at f=6Hz and Θ=2°

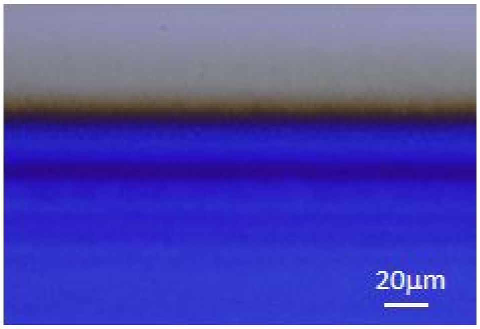

Fig.S5: Morphology of quenched front of film grown at zero frequency. No fishbone structure

has been observed in this case.

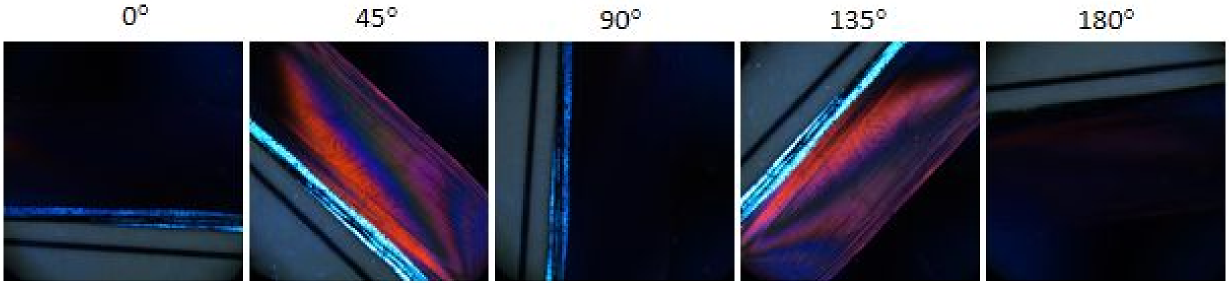

Fig.S6: PM images of an unpatterned P(NDI2OD-T2) film created under static drying condition (f=0) taken at different sample rotation angles. The brightness changes with rotation angles indicate the existing of a preferential chain orientation.

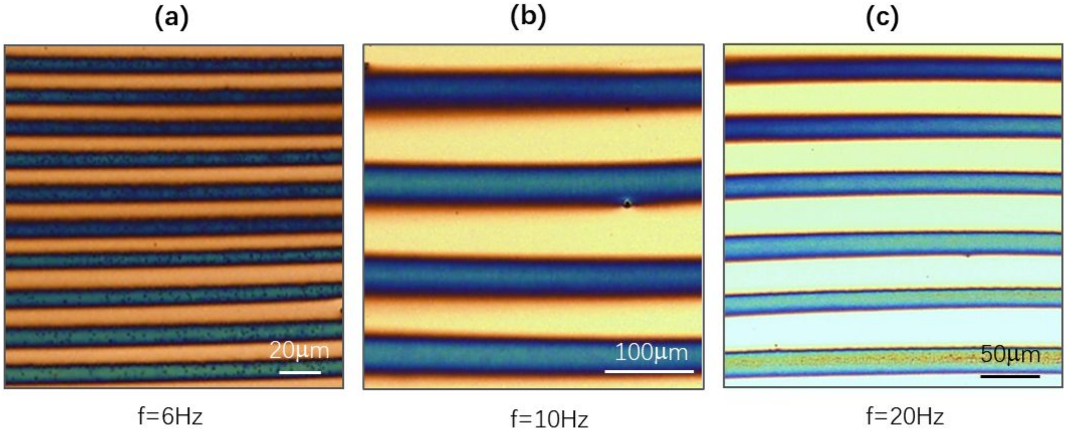

Fig.S7: PDCL mechanism defined P(NDI2OD-T2) line patterns created under different sample-pivoting frequencies (α=25° and Θ=2°). (a) f=6Hz; (b) f=10Hz; (c) f=20Hz

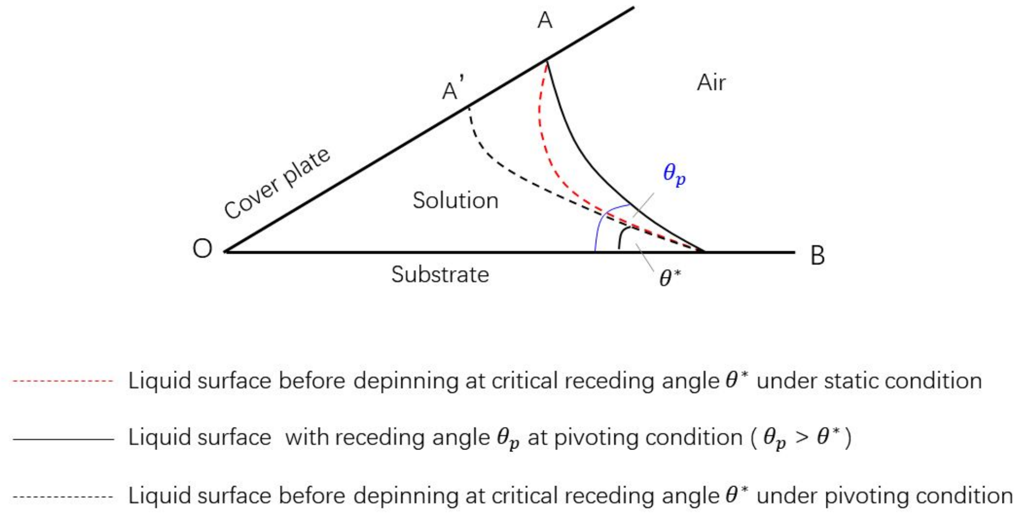

Fig. S8: Schematic illustration of delayed depinning of contact line when sample is dried under pivoting condition. If the sample is drying under a static condition the depinning of the contact line is initiated when the contact angle is reducing to the critical receding angle θ* (red dashed curve). Imagine, at this moment the sample is turned to the pivoting condition, the contact angle will become θ_p instead of θ* due to the vibration (black curve). In this case, to achieve the critical angle θ* further liquid evaporation (A → A′) is required to reduce the contact angle (black dashed line). This will increase the step length of each pinning-depinning process. For simplicity, we assume there is no pinning of contact line on the cover-plate surface in the figure.

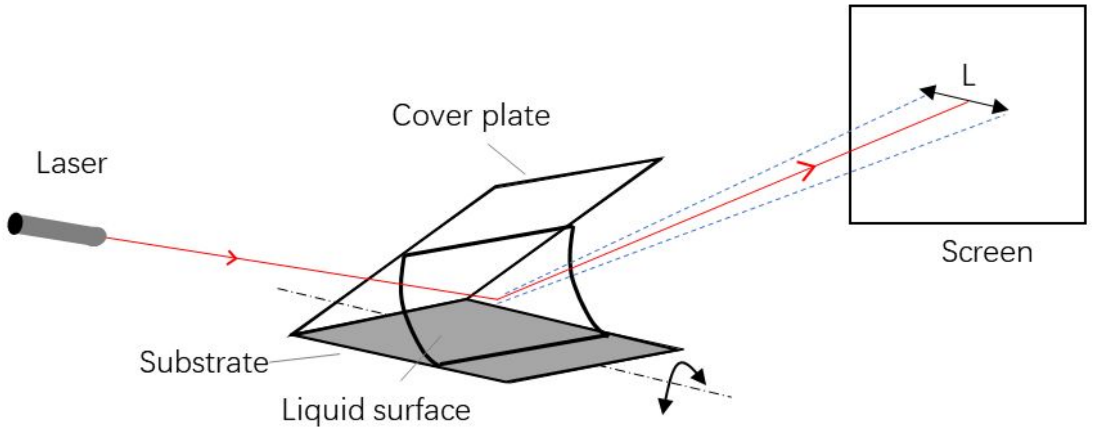

Fig. S9: Schematic drawing of the setup for measuring liquid vibration strength. Laser beam incidents to liquid surface and is reflected to a screen. We measured the range of the beam spot swept over L on the screen and normalized the signal by $= \frac{L - L_{min}}{L_{max} - L_{min}}$.

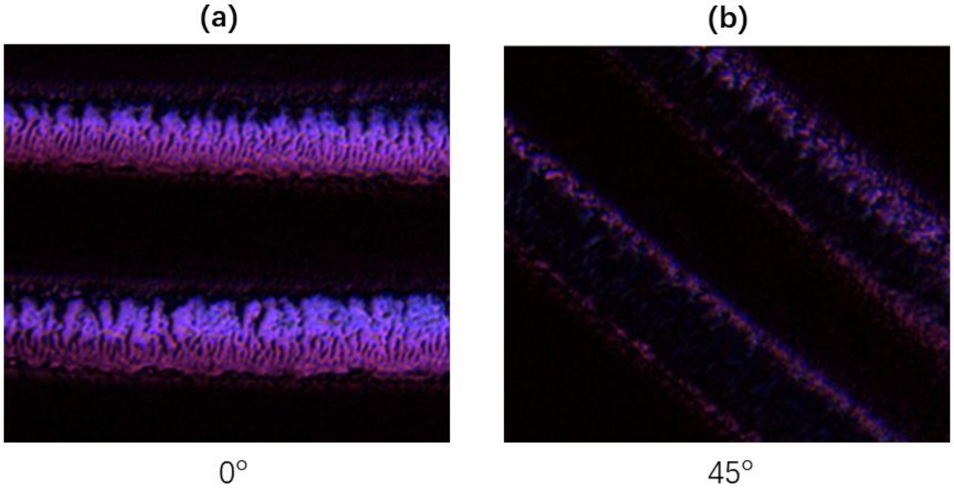

Fig. S10: Polarized microscopic images of PDCL mechanism defined lines taken at different sample orientation angles(θ). (a) For θ=0°; (b) For θ=45°. The birefringence indicates the alignment of polymer chains in the lines.

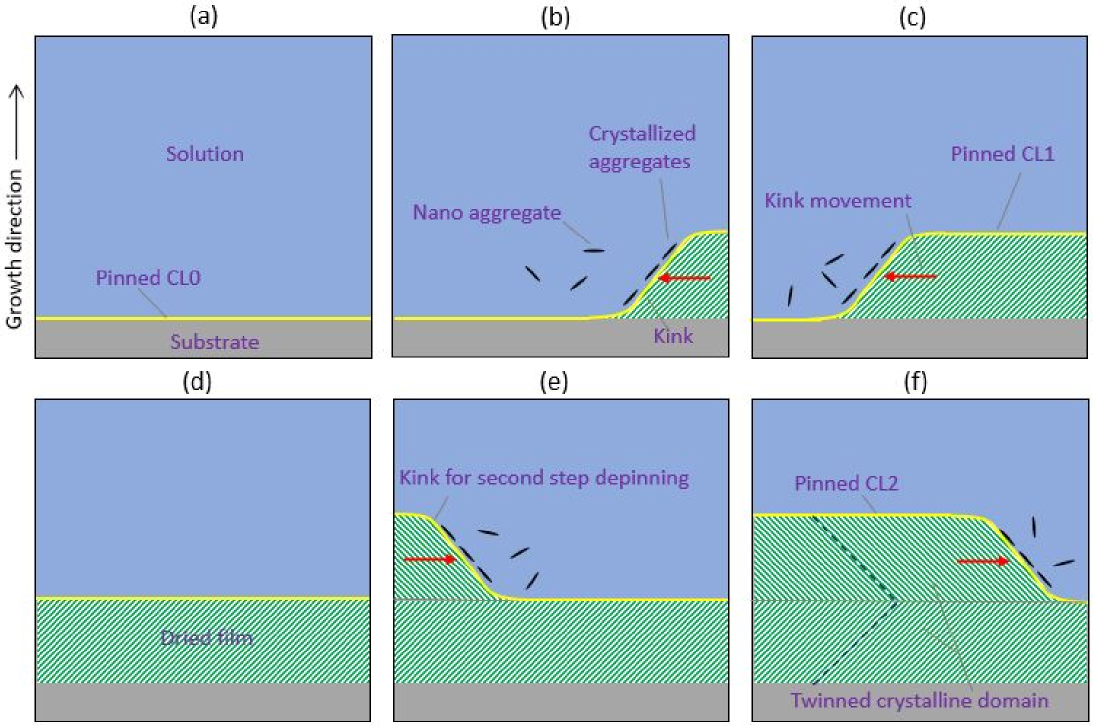

Fig.S11: Schematic illustration of the proposed possible "kink propagation" mechanism to explain the formation of twinned structures. Initially the liquid is pinned at contact line CL0 (a). With progressive solvent evaporation a depinning of the contact line can happen. The depinning can be initiated with formation of a kink (b) and further progressed with the kink-propagation(c) until one step of the depinning completes (d). By completing the first step of depinning at sample edge (or defect) a new kink is formed simultaneously there (e) by an overshoot of the depinning. The newly developed kink will further propagate and continue the depinning (f). During the propagation of the kink polymer aggregates will deposit onto the kink and the dried film become twin-textured. The kink formation is dynamically feasible, because the driving force needed to

overcome the pinning force is drastically reduced as the size of the kink is much smaller than the total contact line, although the kink formation will cause a little local energy enhancement due to the bending of the contact line.
